# Implementation of Mobile Psychological Testing on Smart Devices: Evaluation of a ResearchKit-Based Design Approach for the Implicit Association Test

**DOI:** 10.3389/fdgth.2022.785591

**Published:** 2022-03-18

**Authors:** Tobias Jungnickel, Ute von Jan, Urs-Vito Albrecht

**Affiliations:** ^1^Peter L. Reichertz Institute for Medical Informatics of the TU Braunschweig and Hannover Medical School, Hannover Medical School, Hannover, Germany; ^2^Department of Digital Medicine, Medical Faculty OWL, Bielefeld University, Bielefeld, Germany

**Keywords:** implicit association test, mobile psychological testing, bias, active tasks, iOS, mobile app, ResearchKit-based implementation, programming

## Abstract

**Objective:**

To determine whether a framework-based approach for mobile apps is appropriate for the implementation of psychological testing, and equivalent to established methods.

**Methods:**

Apple's ResearchKit was used for implementing native implicit association test methods (IAT), and an exemplary app was developed to examine users' implicit attitudes toward overweight or thin individuals. For comparison, a web-based IAT app, based on code provided by Project Implicit, was used. Adult volunteers were asked to test both versions on an iPad with touch as well as keyboard input (altogether four tests per participant, random order). Latency values were recorded and used to calculate parameters relevant to the implicit setting. Measurements were analyzed with respect to app type and input method, as well as test order (ANOVA and χ^2^ tests).

**Results:**

Fifty-one datasets were acquired (female, *n* = 21; male, *n* = 30, average age 35 ± 4.66 years). Test order and combination of app type and input method influenced the latency values significantly (both *P*<0.001). This was not mirrored for the D scores or average number of errors vs. app type combined with input method (D scores: *P* = 0.66; number of errors: *P* = 0.733) or test order (D scores: *P* = 0.096; number of errors: *P* = 0.85). *Post-hoc* power analysis of the linear ANOVA showed 0.8 by *f*^2^=0.25, with α = 0.05 and 4 predictors.

**Conclusions:**

The results suggest that a native mobile implementation of the IAT may be comparable to established implementations. The validity of the acquired measurements seems to depend on the properties of the chosen test rather than the specifics of the chosen platform or input method.

## 1. Introduction

### 1.1. Background

Mobile apps running on smartphones, tablet PCs, and other mobile smart devices are not only widely used for social networking and news, entertainment and gaming, travel, shopping, education, or finance, but also for health, fitness, and medical purposes (1).

Compared to apps with an interventional intent related to psychology or psychiatry (2–6), there are currently relatively few mobile apps that aim at supporting non-interventional psychological research [see, for example, (7–9)].

Researchers such as (10) nevertheless emphasize their potential for the field of psychology, be it for psychologists, patients, or the general public. Their utility for research seems apparent considering that, unlike software used on stationary devices, such mobile apps commonly have a narrower focus compared to often more complex desktop applications. This may facilitate their efficiency and reduce development costs. Independent of these factors, programmers still need to be aware of the intricacies of the underlying platform, e.g., related to specific design paradigms.

To help reduce the development overhead, for standardizable processes and tasks, reusable programming frameworks have become the tool of choice independent of the field of application (11, 12). For mobile apps, they commonly provide programmers with convenient, standardized components for the user interface (e.g., survey templates, buttons), methods for accessing a device's sensors, or data management. For research apps, libraries such as ResearchKit (13, 14) for Apple's iOS-based^1^ devices or the ResearchStack library (15) for Android-based devices follow this paradigm. Using these or other frameworks and solutions available for creating apps for research purposes (9) may not only facilitate development, but may also have scientific benefits. These may for example relate to making app-based research more easily reproducible, by allowing researchers to more easily build upon the work of their peers, or, if necessary, to adapt the provided methods to their specific research questions.

Despite the aforementioned advantages, the ResearchKit documentation currently lists only six pre-built “Active Tasks” (i.e., building blocks for ResearchKit-based apps) that can be used in the field of cognition, and thus, in the broadest sense, for psychological research (16). Within this group, for example, the mPower Study (17), evaluates one of the five initially released ResearchKit apps, which applies spatial memory testing in the context of researching Parkinson's disease. Golden et al. (18) use stroop and trail making tests to measure the cognitive effects on caffeine and l-theanine. Finally, Munro (19) analyzes improvements in problem-solving skills using the Tower of Hanoi puzzle for people living with cardiovascular disease during fasting phase.

### 1.2. Objective

The objective of the work presented here is to determine whether and how a framework-based approach is appropriate for the implementation of psychological testing, and equivalent to established methods, such as, for example, web-based approaches. For this purpose, an exemplary, well-established test method, the implicit association test (IAT) was chosen for native implementation on a single mobile platform (namely iOS).

This article describes the underlying methods used for 1. building the native app, specifically its technical aspects and implementation steps, as well as 2. a preliminary cross validation with a web-based installation of the original IAT provided by Project Implicit (20).

A real-world evaluation of the mobile IAT version, using a categorization task similar to the one described here, is however not part of the objective of the presented work and will be described in another publication.

### 1.3. Organization

Since there are several building blocks that form the basis for the study (from data collection to evaluation), the presentation will follow a three-tiered approach.

In the methods part, firstly, the basics of the implicit association test (IAT) will be introduced, along with its structure, setup and the evaluation of the recorded data. This description will also cover essential aspects to consider regarding its implementation on a mobile platform.

Afterwards, the tools and methods used during the implementation phase will be introduced. This part includes a short overview of relevant programming concepts to be used in the native app, specifically regarding data structures and methods provided by Apple's ResearchKit, with an emphasis on those necessary for the actual implementation of the IAT on the chosen mobile platform, i.e., iOS.

The third block will focus on the initial evaluation of the app-based test vs. a web-based test implementation, and will therefore cover aspects related to this evaluation in further detail, using the example of an implementation for evaluating weight-based stigmatization.

Where appropriate, the results section will mirror the breakdown described here by firstly presenting the app based on the described programming methods, and secondly the comparative, comprehensive evaluation of the native and web-based test implementations.

On a side note, while the tests as they are shown in this paper use the English language terminology employed by Project Implicit in their weight stigma related test implementation, namely “fat” vs. “thin,” throughout the text and figures, where applicable, we have tried to use less stigmatizing terms (21) for describing the different weight strata, i.e., “overweight” and “obese” vs. “normal weight” or “lean.”

### 1.4. Scope and Results

Based on ResearchKit, it was possible to build a reusable implementation of the IAT for use on Apple's iOS-based devices. Using this implementation of the IAT, an exemplary mobile app for examining user's implicit attitudes (or bias) toward overweight or thin individuals was then developed for evaluation. For comparison, a web-based implementation of the IAT, using a combination of materials and code provided by Project Implicit (22, 23) was deployed on a Linux-based web server.

Participants that were recruited for the evaluation were asked to work through both versions of the test, once each using the iPad's built-in touchscreen, and another time using a keyboard connected to the device. Thus, each participant had to undergo a total of four tests (in random order). For calculating the scores related to the participants' implicit attitudes, latencies recorded for a user's reaction to specific (combinations of) stimuli were used.

For the actual evaluation, complete datasets for 51 participants could be acquired using the native and web app based versions. There was data for 21 female and 30 participants. On average, participants were 35 ± 4.66 years old.

Both test order as well as the combination of app type and input method exerted a significant influence on the recorded latencies (*P* < 0.001 in both cases). This was however not mirrored for the actual D scores representing the implicit attitude (bias) or the average number of errors vs. input method (D scores: *P* = 0.66; number of errors: *P* = 0.733) or test order (D scores: *P* = 0.096; number of errors: *P* = 0.85). Demographic aspects such as age or gender did not influence the calculated D scores significantly.

*Post-hoc* power analysis of the linear ANOVA showed 0.8 by *f*^2^=0.25, with α=0.05 and four predictors.

## 2. Methods

### 2.1. The Implicit Association Test

In psychology, interest into assessing people's attitudes, behavior patterns, opinions, as well as other constructs in a standardized manner has significantly grown over the past few decades. However, direct questioning of subjects on sensitive topics may result in responses that are more in line with societal expectations than with a person's actual opinions and attitudes. One way to work around these problems is to employ so-called implicit measures. Implicit measures are based on an individual's reactions while performing a series of categorization tasks for specific, contrasting conditions. It is assumed that such tasks will be performed with a higher accuracy and in less time if the presented stimuli that represent the conditions and categories are in line with the person's attitudes toward the topic being evaluated. Roughly speaking, the actual measurement of individual bias, in the form of a differential score, is then calculated from the difference in the response times (latencies) for the contrasting conditions and stimuli that the test subject is asked to categorize (24). A popular test method in this context is the implicit association test (IAT) that was first introduced by Greenwald et al. in the late 1990s (25, 26).

The main reasons for selecting this specific test for our project were that

- it is a simple psychometric method for implicit social cognition, and also allows determining how strongly two complementary concepts (e.g., shown as textual or pictorial representation, such as silhouettes of overweight vs. normal weight people, hereafter referred to as concept 1 and 2) are associated with either of two contrasting attributes (e.g., a set of positively vs. negatively connoted words)
- various (multilingual) sample implementations are available, mainly in digital (web-based) form (20), with sample data sets (and the source code) often being provided (27), which made a comparison of our work to existing implementations feasible, and
- that the IAT is established in the field for gaining insights into the (implicit) attitudes of test subjects related to varying topics [see, for example (28) for a review investigating applications of the IAT toward individuals with various disabilities or (29) for its use in the context of moral concepts].

To the best of our knowledge, there are currently only few native implementations of this specific test on any mobile platform, such as the “Implicit Association Test” app provided both for the Android (30) (last updated in 2014) as well as the iOS platform (31) (last updated in 2013). However, source code for these is unavailable, and subject areas are not configurable. In case of the aforementioned app, only gender-bias is tested.

The iOS platform was selected for the exemplary implementation of a broader approach described here because Apple, who, as the manufacturer, is intimately aware of the platform's specifics, provides ResearchKit, an open source framework specially adapted to this platform (14). Soon after its initial release, ResearchKit already proved its value for research in projects of various working groups (32). Frameworks available for other (mobile) platforms commonly do not benefit from a similar degree of integration with the respective platforms.

#### 2.1.1. Basic Structure of the IAT

As defined by (33), there are seven blocks an individual has to work through when an IAT test is administered. There are (shorter) blocks where a test subject may practice the classification tasks (i.e., *B*_1_ to *B*_3_, as well as *B*_6_, with 20 trials each and *B*_5_ that may vary between 28 trials for the US IAT (34) and 40 trials for the German IAT (35), as well as (longer) test blocks of 40 trials each. While *B*_1_, *B*_2_, and *B*_5_ are sorting blocks only presenting terms of one category—concept or attribute stimuli—blocks *B*_3_, *B*_4_, *B*_6_, and *B*_7_ present paired terms of both categories. In cases such as our study, where the IAT is administered to multiple individuals, and possibly more than once, it makes sense to randomly assign the order of blocks. This specifically relates to which of the contrasting attribute types or concept is assigned first to the left side, with the order of presentation switched between blocks *B*_1_, *B*_3_, and *B*_4_ vs. *B*_5_–*B*_7_ for concepts, respectively [see Table 1, adapted from (33) for a basic description of the block order for a single participant]. For each individual test run, the side is initially (randomly) assigned for a particular attribute and concept type. For the attribute stimuli, the side is maintained for the duration of the test (e.g., with positive attributes either on the left or right side of the screen). For a larger number of participants, this should prevent a bias being caused by the presentation of certain classes of stimuli on only one side.

**Table 1:** Sequence of trial blocks for assessing two subjects (e.g., overweight vs. lean individuals as concepts 1 and 2).

**Block**	**Number of trials**	**Function**	**Items assigned to left (key) response**	**Items assigned to right (key) response**
B1	20	Sorting practice	Random images or word for concept 1	Random images or words for concept 2
B2	20	Sorting practice	Positive attributes	Negative attributes
B3	20	Pairing practice	Positive attributes + random images or words for concept 1	Negative attributes + random images or words for concept 2
B4	40	Pairing test	Positive attributes + random images or words for concept 1	Negative attributes + random images or words for concept 2
B5	40	Sorting practice (change of sides)	Random images or words for concept 2	Random images or words for concept 1
B6	20	Pairing practice	Positive attributes + random images or words for concept 2	Negative attributes + random images or words for concept 1
B7	40	Pairing test	Positive attributes + random images or words for concept 2	Negative attributes + random images or words for concept 1

#### 2.1.2. Evaluation of the IAT Data

Based on the response times (latencies) recorded in Blocks *B*_3_, *B*_4_, *B*_6_, and *B*_7_, a differential score (short: D score) representing an individual's reaction to the presented stimuli is calculated (33) that represents a user's implicit bias toward either of the two concepts. There are basically six different algorithms *D*_1_–*D*_6_ that can be applied for obtaining this D score. Their choice depends on whether users are provided with feedback (e.g., a red ×) in case of erroneous answers and how the answers that were given too fast to be plausible are handled. Detailed information about this can be found in the literature [e.g., (24, 36)].

For the actual score calculation, there are several external packages and libraries that can be applied to the acquired raw data [e.g., as described in (37–40)].

### 2.2. IAT Implementations Employed in the Study

#### 2.2.1. App-Based Implementation of the IAT: Programming Environment and Employed Concepts

For extending the ResearchKit to include an IAT and building the iOS-based IAT app, the latest version of Xcode was used on an Apple Mac (at the time of the implementation of the app, running Xcode 11.6 on macOS Catalina 10.15), along with the ResearchKit framework (version 2.0). While ResearchKit supports development for both the Objective-C and the Swift languages, it was originally developed in Objective-C, and the latter was also chosen for the purpose of developing the ResearchKit-based IAT test classes. However, the pilot app employing these classes for use in initial testing was implemented using the Swift language, which also allows access to Objective-C based source code.

##### 2.2.1.1. Relevant ResearchKit Elements and Paradigms

ResearchKit specifically supports the development of health-related research apps for iOS (Apple iPhone and iPod touch), iPadOS (Apple iPad), and watchOS (Apple Watch) devices. It was announced and open-sourced by Apple in March 2015 (13). Its source code is available on GitHub under a BSD style license (14) and provides the basic structural and methodical framework for apps that are used in (medical) research.

Tasks are the basic element study participants are confronted with when using a ResearchKit-based study app. They lay the foundation for common activities such as obtaining and handling consent, as well as executing surveys (questionnaires) and active tasks for (touch input or sensor-based) data collection. As this paper describes the functional and technical implementation of the IAT in ResearchKit, it will focus on active tasks employed in this context, while also briefly touching on the basics of consent acquisition and performing surveys.

A basic, (ordered) task object^2^ defines the processes of a specific task at hand. The task object determines the order in which the individual steps are performed, either in a fixed or adaptive flow (depending on previous results), and provides methods for indicating progress.

Tasks are divided into steps^3^, roughly corresponding to a single screen each, that take care of presenting information to the user, as well as data acquisition for the respective step. While many of the available steps either present data or ask users to (manually) enter data in answer to one or more questions^4^, there are also so-called active steps^5^ that enable (automatic) data collection.

There are basically three modules for such tasks that can be adapted to the specific research question:

- The “consent” module is meant to be used for obtaining informed consent when an app is initially started. This includes methods for providing general information about the study (e.g., purpose, type, and amount of data gathered, rationale), determining individual eligibility, etc. The provided consent templates have to be set up by the developer depending on the specifics of the respective study.
- The “survey” module provides templates for confronting users with a sequence of questions, and there can either be a single or multiple questions per screen, with numerous answer types being allowed (e.g., multiple choice, text, or number input). It is possible to make the sequence of questions adaptable by branching into more detailed sub-questionnaires or skipping certain questions depending on answers given in previous steps.
- Active tasks (16) enable researchers to gather data that differs from what can be acquired in surveys, and these are the main foundation for the ResearchKit-based IAT. They can collect data from multiple sources such as different device sensors, audio input, or even data acquired from the heart rate sensor. There are already a number of tasks that were developed for use in research, e.g., related to gait and balance (using motion data), as well as some psychological tests such as spatial memory (17), stroop, or trail making tests (18).

How tasks are managed in the user interface (and their results are handled), is defined by task view controllers^6^. For each step, there are special view controllers^7^ for handling the workflow. Overall, the task view controllers take care of handling results obtained in the steps, and these are not only accessible once a task has completed, but, if necessary, also while it is still in progress.

Specifics of the mobile, app-based implementation of the IAT will be described in the relevant part of Section 3.

#### 2.2.2. Web-Based Implementation

**Figure 1:**
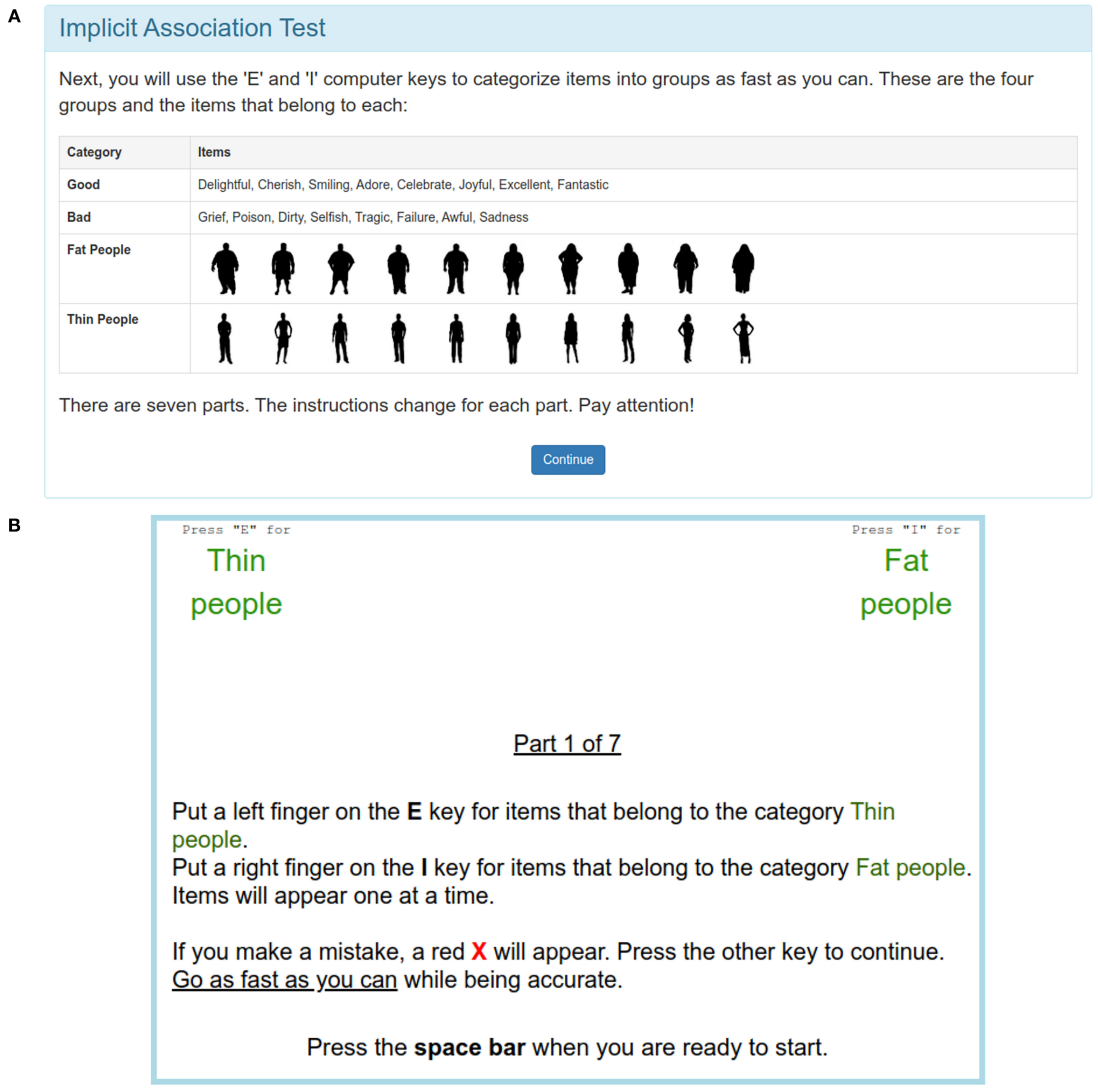
Web-IAT: **(A)** Introduction for the weight-based IAT (cropped) and **(B)** first classification task (English language version for illustration purposes).

For comparison between the mobile IAT and the original, web-based version as used by Project Implicit (20, 27), a local installation of the Web IAT was prepared, based on a combination of the experiment materials provided for the Weight IAT, as provided on (23), as well as one of the examples given for the minno.js-based minimal server available at (22). The adapted version that was used corresponded to the Weight IAT instance provided for users in the United States, based on silhouettes of overweight as well as normal weight individuals, along with positive or negative terms. The web-based app was deployed on a Linux server (at the time of the evaluation, Ubuntu Server 16.04 LTS, using the Apache and PHP packages supplied with this release). As all potential participants were native German speakers, in contrast to the examples shown in Figure 1, the web IAT employed in our study used the descriptions and terms of the German IAT, and wordings between both the app and web-based versions were aligned in order to prevent potential bias in this regard.

### 2.3. Comparative Evaluation of Both Approaches

Potential participants for the comparative evaluation were recruited from a professional and private circle and were asked for their informed consent. Participants were given the opportunity to withdraw their participation at any time.

The evaluation setup itself consisted of four IAT test that were to be performed on the provided iPads:

- The native IAT app, based on the aforementioned ResearchKit classes, one test being administered with an external keyboard, one using the device's touch screen.
- A web-based IAT implementation using the JavaScript and PHP constructs provided by Project Implicit (34), again one test each being applied using the keyboard and touch screen input methods.

In both the native as well as the web-based version, for the tests relying on the touch interface, there were two buttons, one on the left and one on the right-hand side of the display, with the stimuli (terms as well images) appearing centered between the two buttons. The respective category assigned to each button was shown in close proximity, above the button itself.

To stay consistent with the Web versions provided by Project Implicit, for those test runs relying on keyboard input, the “E” key was used in lieu of the left button, and “I” had to be pressed for items assigned to the right-hand category.

All four tests were performed on Apple iPads (8th generation, 10.2-inch display) running the latest operating system version (at the time of the study, iPadOS 14.0.1). The keyboard model used—both for the native app and the web version—was an Apple Smart Keyboard connected via Apple's Smart Connector. For the native app, results were initially only stored locally on the respective iPads, while for the web-based version, results were kept in a protected directory of the web server.

#### 2.3.1. Study Procedure

The participants were assigned a random identifier to be able to compare the four test variants on an intraindividual basis. This identifier was entered manually per test and participant. The order of tests—native or web-based app with either touch screen or keyboard input—was randomized for each participant in order to minimize bias, e.g., due to higher latencies, decreasing concentration, and thus possibly increasing number of erroneous categorizations for repeated testing due to fatigue after repetitive execution of the tests.

For each participant, all four tests were performed on a single day, with a short break (usually around 1 min) between the tests, and altogether, the test sessions did not require more than 30 min per participant.

The participants were also asked to fill out an additional online survey (using a SoSciSurvey installation at the authors' university) using their individual, randomly assigned identifier. The questionnaire presented in this survey was comprised of demographic questions (sex, education, and age) as well as questions related to weight (i.e., explicit preference between overweight or normal weight persons). Participants were also asked about their individual height, weight, and personal interest into the topic of obesity or diabetes).

Answers to all of the questions were optional (in case that any of the participants felt uncomfortable providing any of the answers), and filling out this survey took <10 min per individual.

#### 2.3.2. Evaluation of the Study Data

The datasets acquired using the web and native app based implementations of the IAT were evaluated using R (version 4.1.2) for both descriptive as well as statistical analyses.

For the description of the study population, it was initially decided to stratify by gender, which seemed the most promising due to the study population's relative homogeneity regarding other demographics. In literature, various sociodemographic factors are often associated with influencing an individual's body weight perception or predisposition to stigmatizing individuals based on their weight (41, 42) [while there are other authors that refute this claim at least for some factors; (43)]. As the recruited participants hailed from similar backgrounds and were largely of similar age, gender was the most obvious demographic factor we deemed to potentially have an effect on (explicit or implicit) attitude regarding body image and weight.

To determine whether there were any significant differences in means at different points in time or between the different app and input types, aside from descriptive analysis, ANOVA testing was applied where appropriate. A *post-hoc* power analysis of the linear ANOVA was conducted using G*Power [version 3.1.9.6, (44)].

Altogether, the statistical analysis aimed at comparing the native, ResearchKit-based version of the app with its web-based implementation using both touch screen and keyboard-based user interactions. To determine whether the app type and input method or even the order in which the four combinations had been applied influenced the results, this part of the analysis was applied to both methods of stratification. More specifically, the evaluation focused on the influences of app type and input method as well as test order on either the D scores that were obtained as well as the latencies that were recorded for each trial.

## 3. Results

### 3.1. Mobile Implementation of the IAT

As, to the best of our knowledge, there were no implementations of the implicit association test (IAT) for ResearchKit-based iOS apps when the project was initially planned, it was decided to fork ResearchKit's repository on GitHub (14) and to add the IAT related functionality to this fork. The resulting code, after integration of the IAT, is available on GitHub (45).

#### 3.1.1. Forking the ResearchKit Framework

The design of the presented IAT implementation closely follows the currently provided United States (English) version of the Project Implicit web version (20) as closely as possible. It has however been adapted from the keyboard input used in the web-based version to employ the touch interface available on mobile devices. Ideas on how to better adapt the implementation to the mobile platform will be explained as part of the development related considerations that are presented in the discussion.

The mobile IAT implementation is comprised of seven components. Four of these, implementing the step, content view, view controller and result objects with specific adaptations to the IAT test's requirements^8^, follow the common class structure established for active steps in ResearchKit. The additional three components were designed for providing instructions on how to perform the test, or for specific aspects of the presentation of the IAT^9^. There is also a predefined active task for the IAT steps, extended from the ORKOrderedTask object, which is used to initiate the test process.

Since ResearchKit apps commonly only take care of data acquisition and do not include any algorithms for evaluating the acquired data, it was decided to only include a basic implementation of the D score calculation in our study app. This functionality is however not part of the ResearchKit classes upon which the study app is based. For the app, the calculation was however included to be able to provide participants with feedback about their score if so desired. Similar to our study app, developers making use of the provided IAT classes will also need to implement this functionality in separate parts of their app should they decide provide score related feedback instead of solely evaluating the data at a later stage.

For illustration purposes, the screenshots shown in the following paragraphs are in English language and use silhouettes of overweight people for the first and individuals of slim to normal stature for the second concept stimulus, but these settings can of course be adapted, e.g., to support random assignment of the chosen stimuli to either side in the actual study app used in the evaluation.

##### 3.1.1.1. Look and Feel of the IAT

**Figure 2:**
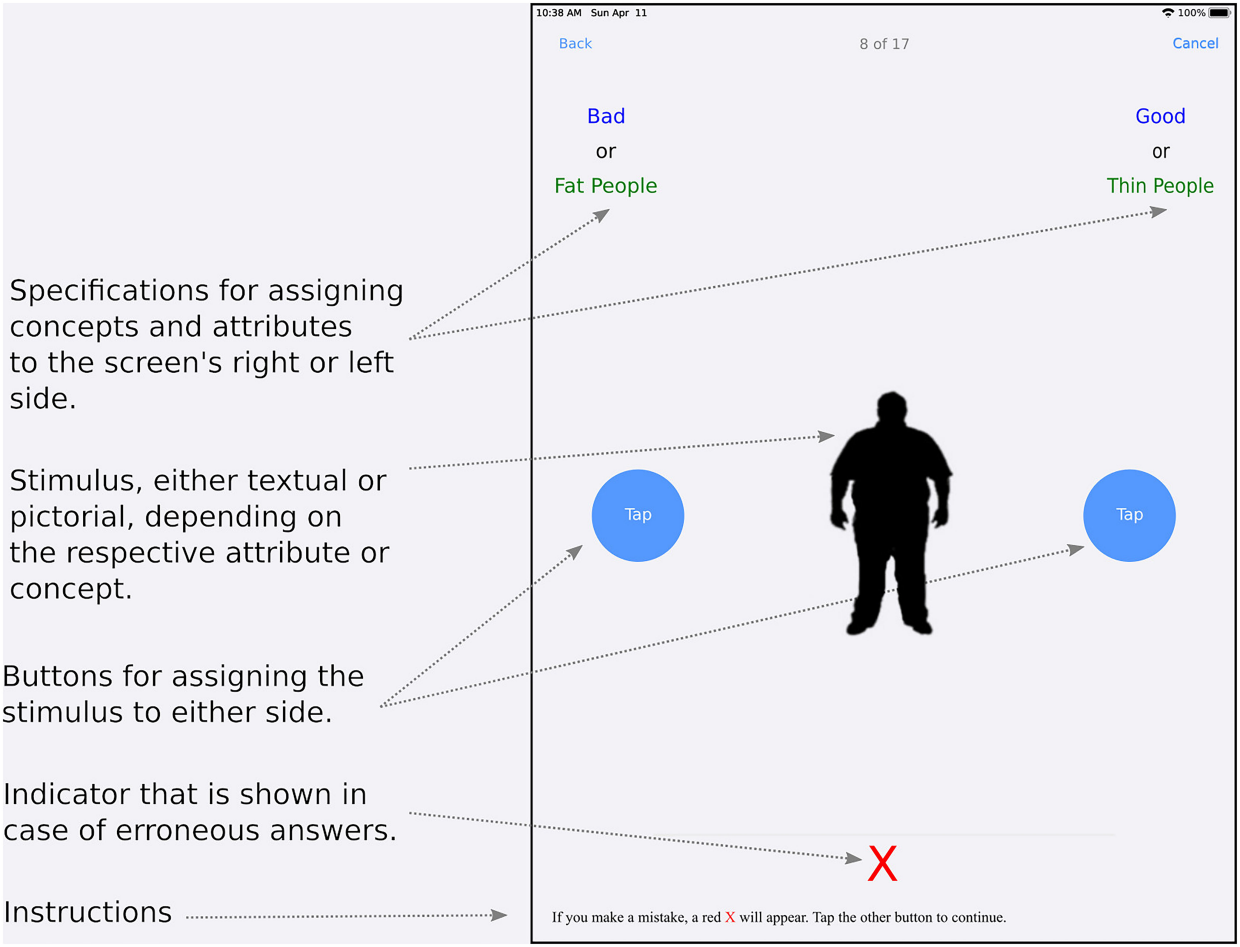
Schematic for the basic layout of the IAT View for a ResearchKit-based app.

An ORKImplicitAssociationContentView, subclassed from ResearchKit's ORKActiveStepCustomView class—which serves as the basis for custom views in active steps—provides the visual interface for an IAT trial (Figure 2). It defines two containers (based on UIView) for items—one in the upper left and one in the upper right corner—each containing one label for the first or only item, and, in cases where concepts and attributes are paired, additional labels for a divider as well as the second item. The first label displays either the identifier for the respective attribute or concept, i.e., “positive” or “negative” attribute or “concept 1” or “concept 2” in the sorting phase, or the category name for attributes—“positive” or “negative”—in the pairing phase.

In the latter case, the second label shows the category name for concept stimuli—“concept 1” or “concept 2”—while the dividing label displays the term “or” to instruct the user to touch the button on the appropriate side when either a corresponding attribute or a concept stimulus being displayed in the corresponding part of the screen (Figure 2). Both the second label as well as the divider are hidden in sorting phases. As implemented in the study, attribute category names are always colored blue, while those for concepts use green color. Dividers (“or”) are always shown in black color.

In the view's center, there is a term container (again, based on UIView) containing a label and an image^10^ showing either (exclusively) the current attribute or concept stimulus in the trial. Another container hosts round tapping buttons (ORKRoundTappingButton) on either side of the screen. As an alternative, when using a keyboard, the keys “E” for the left, respectively “I” in place of the right tapping button can be used. Initially, a label indicating that one of the buttons must be touched to start the test is shown at the screen position where later on, the term label or image will be shown. In the view's lower part, users are informed that an error indicator in the form of a red × will be displayed in case of any misclassifications, indicating the need for reclassification of the current stimulus. In any such case, this error indicator is displayed directly above this hint.

All label elements used in this view are inherited from UILabel class.

To allow ORKImplicitAssociationStepViewController to exert control over the user interface of the IAT, ORKImplicitAssociationContentView provides six methods for external access:

- Firstly, a mode (of type ORKImplicitAssocia-tionMode)—either “instruction” or “trial”—has to be set. In the first case, the term label and image are hidden, while the introductory label is displayed. In test mode, this is the reverse.
- Secondly, the term (NSObject) and its category (either attribute or concept, ORKImplicit-AssociationCategory) has to be specified. The term can either be a string (for an attribute stimulus) or the image of a concept stimulus to be displayed in the trial. The color in which the term is shown depends on the category: Terms representing attribute stimuli are always colored blue and those for concept stimuli are green. This corresponds to the coloring of the category names in the view's upper left and right corner.
- To specify the names for attribute and concept categories, two methods are provided.
  - The first method defines the names of the categories on both sides (NSString) as well as the category type (ORKImplicitAssociationCategory, either attribute or concept). The type is used to choose the appropriate color (blue or green).
  - The second method determines which labels are shown first and second (separately, for both sides of the view). There is no need to specify the corresponding categories to define colors, as the initial labels always show the attribute category names (blue) while the second labels show the concept category names (green).
- The fifth method can show a red × in the event of misclassifications.
- Finally, it is possible to disable the presented classification buttons once the user has correctly classified the current stimulus. This is done to prevent reactions to inadvertent additional taps on the buttons.

There are also two tapping button objects that are programmatically accessible from outside the ORKImplicitAssociationContentView in order to be able to react to taps in the ORKImplicit-AssociationStepViewController.

##### 3.1.1.2. Control of the IAT's Blocks

An ORKImplicitAssociationStepViewController (derived from ORKActiveStepViewController) controls the logic for an IAT block and structures the lifecycle for the so-called active steps employed to represent the blocks of the IAT. On startup, it sets the mode of the view (ORKImplicitAssociationContentView) to “instruction,” and for sorting or pairing blocks, passes the category names (ORKImplicitAssociationCategory) to be displayed on the top left and right corner to it accordingly.

For any button taps executed by a user, the procedure remains identical within the currently running IAT session. Once the correct choice has been made within the last trial of the respective block, the current step is completed and the next step within the task is started. The structure for each trial is the following:

- The trial enforces the ORKImplicitAssociation-ContentView to hide the error indicator (i.e., the red ×), sets the mode (ORKImplicitAssociationModeTrial) to trial to show the term, and hides the start label. It also passes the term and its corresponding category (ORKImplicitAssociationCategory, attribute or concept) to be displayed, and activates the buttons in order to allow taps. Note is also taken of the point in time at which the trial was started.
- As soon as a button is tapped, the view controller checks if this event took place on the correct side of the display. If not, the ORKImplicitAssociationContentView is instructed to show the error indicator and to log that an error has been made within the respective trial. This is repeated as long as the user keeps making an incorrect choice. Once the expected answer has been given, the ORKImplicitAssociationContentView is instructed to disable the buttons and to hide the error indicator. An ORKImplicitAssociationResult is then created to save the time span (latency) between when the term was initially shown and the point in time when the correct answer was given. The trial code of the correct term as well as the pairing of the categories, and whether the answer was initially incorrect are recorded as well.
- Finally, the process is started once again for the next trial.

##### 3.1.1.3. Keeping Track of Results

An ORKImplicitAssociationResult (derived from ORKResult) holds the results per trial. It is meant to keep track of the overall latency (i.e., the time between the initial presentation of a stimulus until the correct answer has been given). The trial code, for identifying on which side the term—i.e., attribute or concept—correctly matched, as well as the (concept and/or attribute) pairings employed on either side of the view for that trial are also included, as is whether the initial classification for that trial was correct or not.

To enable serialization of the IAT's results into JSON, the ORKESerialization class was extended. This base class is available within the ORKTest project that is provided within ResearchKit, and is meant to test functionality during development. For the purposes described here, this added functionality includes being able to take note of the latency as well as the trial code, the pairing of the categories, and information about whether the user's initial reaction to the respective stimulus was correct.

##### 3.1.1.4. Step Objects for the IAT

ORKImplicitAssociationStep forms the basis for active task steps used in the implicit association test and is derived from ORKActiveStep. Used to represent the blocks of the IAT, it manages the respective number of trials of the block in the form of an array (NSArray of ORKImplicitAssociationTrial) and also keeps track of whether the respective block is a sorting or pairing block (ORKImplicitAssociationBlockType).

##### 3.1.1.5. Keeping Track of a Trial's Information

Objects of type ORKImplicitAssociationTrial hold the information for the trial within a block. This includes the term to be displayed (either text or an image), the category (ORKImplicitAssociationCategory) of that term (either an attribute or concept), as well as the initial items shown on either the left or right side of the view, containing either the attribute or concept category name (for sorting blocks) or the attribute (for pairing blocks). For pairing blocks, the concept category name used for the left and right side is always stated. In addition, the correct term (ORKImplicitAssociationCorrect) representing the left or right attribute—for attribute sorting blocks—or the first or second target on the left or right side, respectively, are specified. There is also a computed property returning an identifier (ORKTappingButtonIdentifier) for indicating whether the left or right button needs to be chosen for giving the correct answer.

##### 3.1.1.6. Supporting the UI Design

ORKImplicitAssociationHelper defines the colors to be used for displaying attribute (blue) and concept (green) names, button side names (left or right in light blue), and the red error indicator symbol ×. These colors are not only used in the active steps of trials, for both sorting and pairing blocks (ORKImplicitAssociationContentView), but also be for attribute and concept instructions ORKImplicit-AssociationCategoriesInstructionStep as well as for the instruction pages (ORKInstructionStep) before each block. This will be explained later on.

ORKImplicitAssociationHelper also contains a method to convert a text that may contain XML-based tags (e.g., <attribute>, <concept>, …) into an attributed string, which in turn can be displayed in a view. This is provided in order to simplify the color design of instruction pages for developers.

##### 3.1.1.7. User Instruction

An ORKImplicitAssociationCategoriesInstructionStep can display all attribute stimuli and concept stimuli in a tabular view once an IAT has been started. It is subclassed from ORKTableStep and has methods to pass the category names for the two attributes and both concepts as well as the terms for each category. Terms can either be texts for word attribute and concept stimuli or image file names for image concept stimuli (kept in arrays of the appropriate object types).

##### 3.1.1.8. Defining the Order of Things

ORKOrderedTask+ORKPredefinedActiveTask is an extension of ORKOrderedTask to define the steps (ORK-ActiveStep and ORKStep) for an active task (implementing the ORKTask protocol) to be presented from a task view controller (ORKTaskViewController).

Two functions with partly different parameterizations were added that allow creation of an IAT task depending on the respective requirements. Both implement three parameters that are configurable for all active tasks:

Firstly, there is a textual identifier for the task, and secondly an optional description for the data collection's intended purpose. The third parameter represents predefined task options (ORKPredefinedTaskOption), e.g., to exclude instruction and conclusion steps, or to prevent data collection from the device's sensors (such as accelerometer, location, or heart rate data). Both functions also allow to pass the required IAT specific parameters, e.g., the two attribute and two concept category names, as well as the terms (texts for word attribute and concept stimuli or images for image-based concept stimuli) for each category (provided as arrays of the appropriate data types).

The second of the two functions differs from the first in that it allows to pass additional parameters, such as the number of trials for each of the seven blocks. It also makes it possible to enable or disable randomization of concepts and attributes to either side. If not specified otherwise, blocks 1, 2, 3, and 6 are set up with 20 trials, block 4 and 7 are set up with 40 trials and block 5 is set up with 28 trials, as in the Project Implicit US Web IAT (34). Also, per default, the sides on which attributes are displayed are not randomized—meaning that the first attribute is always presented left while the second attribute is presented right—while the concepts are randomized to either side; also see Table 1 above. All randomizations are programmatically based on the RC4 cipher (Rivest Cipher 4).

A complete test run is constructed as follows: First, the concepts for blocks 1 and 5, as well as the attributes for block 2 are randomly selected from the respective sets of available stimuli. The numbers of chosen stimuli correspond to the numbers of trials for each of the blocks (see Table 1). Then, for each of the blocks 3, 4, 6, and 7, attribute and concept stimuli are randomly chosen so that within each block, the numbers for the contrasting attributes and/or stimuli are in balance.

Next, the steps of the IAT, managed by the IAT active task, are constructed. For this purpose, first, an overview of all attribute and concept stimuli is created, as well as an introductory instruction for the IAT^11^. Both steps can be skipped if an option is passed to the IAT active task method that specifies the exclusion of instruction steps (ORKPredefinedTaskOptionExcludeInstructions). Secondly, for each of the seven blocks, one step object is created (ORKImplicitAssociationStep), and the numbers of trials are added by using the ORKImpli-citAssociationTrial with the terms that were previously selected as mentioned above. Before each block, an introductory instruction step (ORKInstructionStep) is added to provide information about what is expected in the respective block. These instruction steps can also be included by passing the option to exclude instructions. Lastly, a final step is added for informing users about the completion of the IAT task^12^. Both methods finally return the created IAT active tasks that can be presented to users as an ORKTaskViewController within the app.

#### 3.1.2. Integrating the ResearchKit-Based IAT in a Project

To build an IAT app for the iOS or iPadOS platform, the ResearchKit-based IAT elements as they were described in the previous paragraphs can be employed in two different manners, firstly by using a predefined IAT active task or, secondly, by specifying the IAT steps manually.

##### 3.1.2.1. Using the Predefined Active Tasks

As described for ORKOrderedTask, a predefined active task can be created by calling one of two methods. This provides the full IAT implementation, with its seven blocks and the corresponding default (or adapted) numbers of trials for each block, as well as the (optional) instruction and completion steps. Both methods return an ORKTaskViewController (subclassed from of UIViewController) that can be shown in any iOS or iPadOS application.

The results of the IAT can then be obtained from the task view controller's result property (of type ORKTaskResult, subclassed from ORKCollectionResult), which in turn holds the results in its results property—an array of ORKStepResult each containing all its ORKImplicit-AssociationResult objects. The step results for each IAT block are identified by implicitAssociation.block1 to implicitAssociation.block7.

##### 3.1.2.2. Using the Active Steps Manually

The IAT active step may also be used separately as a step inside any task a programmer decides to build. An ORKImplicitAssociationStep has to be initialized with an unique identifier. A block type (ORKImpli-citAssociationBlockType) can be assigned to the step to distinguish between sorting and pairing blocks. Finally, an array of trials (for the trials within the block) (ORKImplicitAssociationTrial) has to be assigned to the trials property of the respective step. The results can be obtained in the same manner as described for the predefined active task, by identifying the step results via the identifiers that were specified.

#### 3.1.3. An Example of a Mobile IAT on the iOS Platform

The following paragraphs and figures give a short overview over an actual (English language) implementation of the IAT task predefined in ORKOrderedTask, specifically targeting weight bias (i.e., normal weight vs. overweight individuals). Text shown in italics indicates instruction and completion steps that can be omitted by passing the appropriate options.

**Figure 3:**
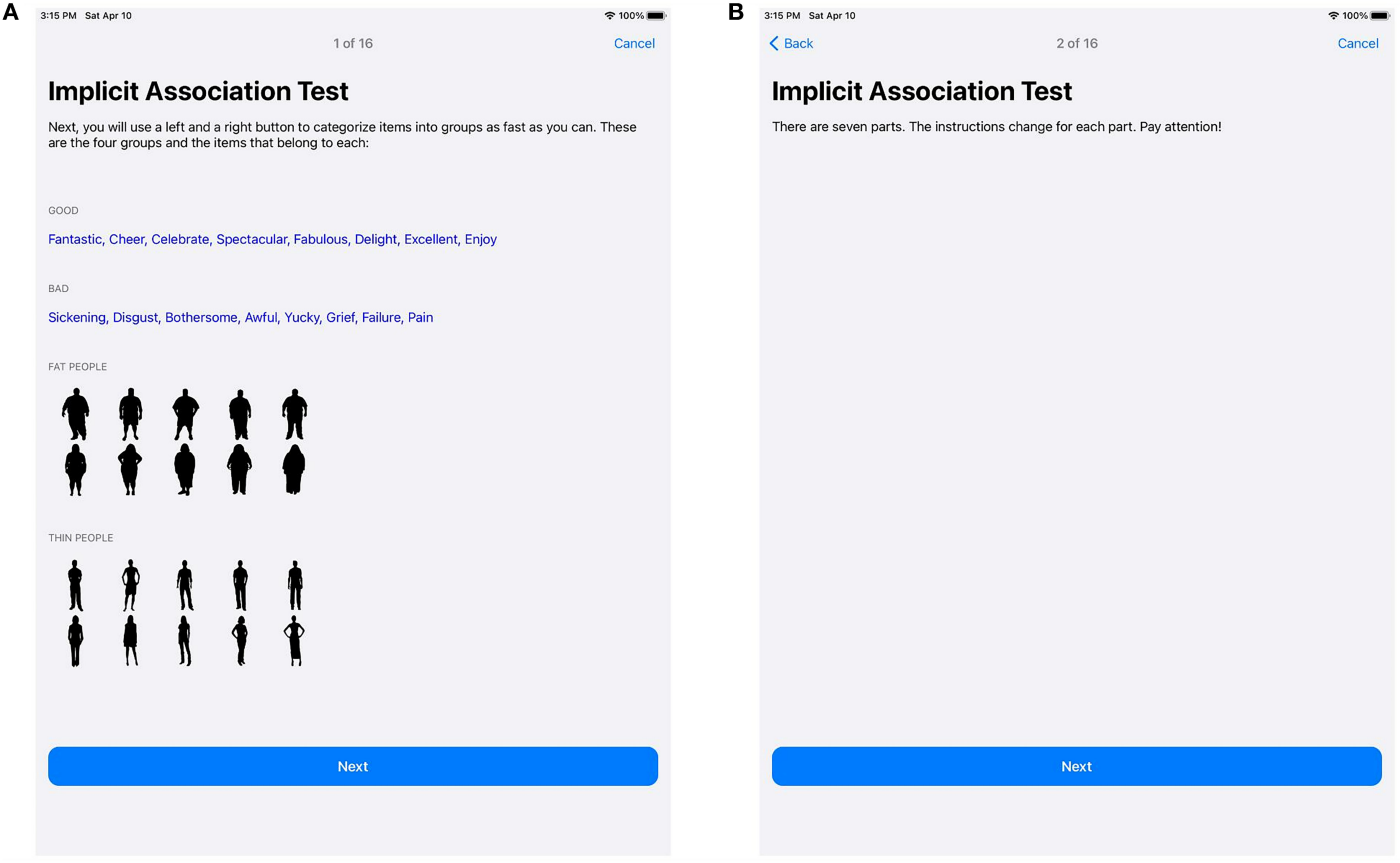
Introduction to the concept and attribute stimuli **(A)**, as well as the basic structure of the IAT **(B)**.

Figure 3 shows the introduction for the IAT active task. Figure 3A introduces the concept stimuli and attribute stimuli for the IAT, while Figure 3B informs users about the structure of the IAT itself and reminds them to stay attentive.

**Figure 4:**
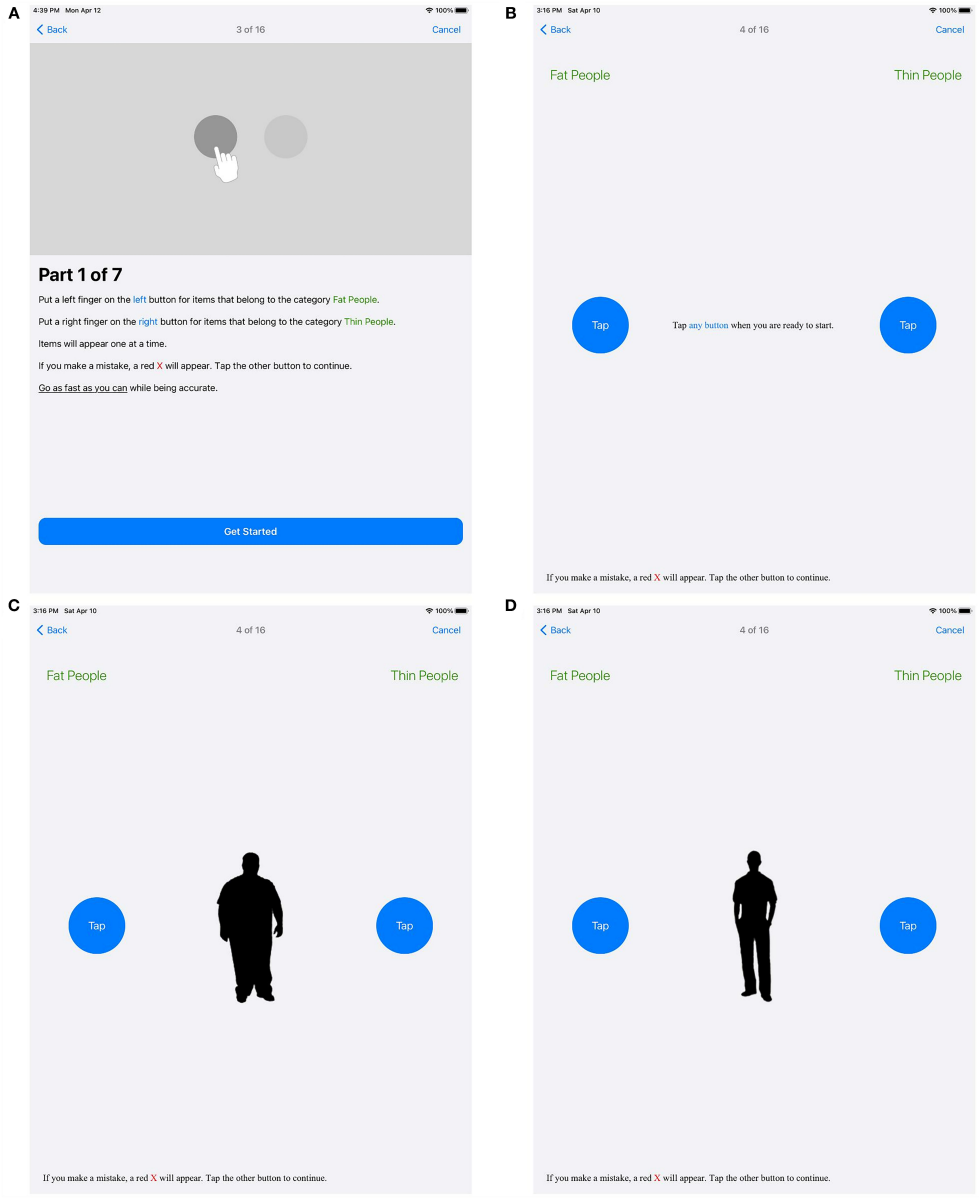
Block 1. Explanations on how to perform the test **(A)** and an information screen **(B)** are displayed before the first test block. During the test, the stimuli are shown in random order **(C,D)**.

In Figure 4, the first block of the IAT is demonstrated. Figure 4A introduces the block with the left button to be tapped for concept stimuli representing overweight individuals, and the right button to be tapped for concept stimuli depicting normal weight individuals, along with general information about the task. Figure 4B shows the information screen just before the test block begins. Terms representing silhouettes for overweight or slender people are randomly presented, as is illustrated in Figures 4C,D.

The second block is implemented similarly, albeit this time, using positive and negative connoted textual stimuli, to be classified as either “good” or “bad,” in place of the silhouette images.

The third block combines the categorization tasks of the previous blocks: Here, the left button is to be tapped if either “good” attribute stimuli or silhouettes of “overweight people” are shown, while a tap on the right button is expected for either “bad” attribute stimuli or silhouettes representing individuals of normal weight. Again, the order in which the stimuli are shown is randomized, and care is taken to use the same quota (i.e., 5 per kind) for each type of stimulus.

Block 4 is similar to block 3, but, as specified in Table 1, uses a larger number of trials (40 instead of 20). Again, the number of trials per type of stimulus is balanced, and the actual order in which they are presented is chosen randomly.

Block 5 essentially uses the same configuration as the first block, the difference being that the sides for categorizing “overweight people” and “normal weight people” concept stimuli are swapped. Also, there are 40 trials in Block 5.

Blocks 6 and 7 correspond to blocks 3 and 4, albeit with the assignment of the concepts to the left and right sides being swapped.

Finally, on the last screen, it is possible to thank users for their perseverance in finishing the IAT. Feedback about the results of the test should be provided in other parts of the app, after the actual IAT test has concluded.

The ResearchKit-based classes described above were employed for constructing the IAT app used in the study. This study app made use of silhouettes of overweight and normal weight individuals for the concept stimuli [as they were provided by Project Implicit, (22, 23)], as well as a number of terms with positive and negative attributes.

### 3.2. Comparison Between the Native, ResearchKit-Based IAT Version, and a Web-Based Implementation

#### 3.2.1. Demographics of the Participants

Participants were recruited from a circle of colleagues and friends. While originally, there were 56 participants, full data sets were only available for 51 individuals (see Table 2). For five participants, either answers related to demographics or parts of the test data were missing. Overall, those who participated were on average 34.9 (sd = 4.7) years of age (with the 21 female participants being slightly, albeit only insignificantly younger than the 30 males, *P* = 0.392), and there were only insignificant differences between the two genders regarding their education (*P* = 0.448). With respect to body mass index, the differences between both groups were insignificant (BMI value: *P* = 0.092). Interest in the topics of diabetes and adipositas significantly differed between both genders only when looking at the data in its original five point scale (*P* = 0.045), and this was largely due to the reversal in proportions between the “not at all” and “less” interested strata between both groups. Rescaled to “not interested,” “neutral,” and “interested,” there were however only negligible differences (*P* = 0.877).

**Table 2:** Gender specific differences regarding overall demographics as well as D scores (representing implicit ratings) for the four test variants.

		**Female (*N* = 21)**	**Male (*N* = 30)**	***P*** **-value**
Demographics	**Age (years)**			0.392^a^
	Mean (SD)	34.6 (4.0)	35.1 (5.1)	
	Range	24.0 to 39.0	22.0 to 42.0	
	**Education**			0.448^b^
	Secondary school certificate	4 (19%)	2 (7%)	
	High school diploma	2 (10%)	6 (20%)	
	University education w/o degree	1 (5%)	0 (0%)	
	Bachelor	5 (24%)	4 (13%)	
	Master's degree	4 (19%)	7 (23%)	
	Diploma	2 (10%)	8 (27%)	
	Doctorate	2 (10%)	2 (7%)	
	Other	1 (5%)	1 (3%)	
	**BMI**			0.092^a^
	Mean (SD)	23.8 (4.0)	25.7 (4.2)	
	Range	18.7 to 36.3	20.3 to 38.0	
	**Interest (diabetes and obesity, rescaled)**			0.596^b^
	Not interested	16 (76%)	23 (77%)	
	Neutral	3 (14%)	6 (20%)	
	Interested	2 (10%)	1 (3%)	
	**Explicit attitude**			0.312^b^
	Strong pref: thin to overwt	0 (0%)	2 (7%)	
	Pref: thin to overwt	2 (10%)	7 (23%)	
	Some pref: thin to overwt	9 (43%)	12 (40%)	
	Like both equally	9 (43%)	9 (30%)	
	Some pref: overwt to thin	1 (5%)	0 (0%)	
Implicit ratings (D scores)	**Native app + keyboard**			0.213^a^
	Mean (SD)	−0.49 (0.36)	−0.62 (0.41)	
	Range	−1.12 to 0.27	−1.38 to 0.49	
	**Native app + touch screen**			0.041^a^
	Mean (SD)	−0.54 (0.34)	−0.74 (0.35)	
	Range	−1.20 to −0.05	−1.35 to 0.01	
	**Web app + keyboard**			0.491^a^
	Mean (SD)	−0.56 (0.31)	−0.62 (0.45)	
	Range	−1.24 to −0.06	−1.50 to 0.31	
	**Web app + touch screen**			0.632^a^
	Mean (SD)	−0.63 (0.36)	−0.58 (0.39)	
	Range	−1.49 to 0.26	−1.39 to 0.20	

a *Kruskal-Wallis rank sum test*.

b *Pearson's Chi-squared test*.

Neither were there any major differences in explicit or implicit ratings between female and male participants (see Table 2). Only in case of the numeric D score value for the native app being used with the touch screen-based interface was *P* significant (*P* = 0.044), but even for this case, there were no relevant differences considering the D score category (*P* = 0.104). In all other cases, differences in ratings between both genders were negligible (i.e., *P* > 0.05 in all cases).

Overall, for the participants included in this evaluation, the influence of gender on the attitudes (Table 2) regarding personal preferences of normal weight to overweight individuals seems negligible. For other demographic factors, due to the relative homogeneity of the participants, there was insufficient data to make a reliable assessment. A decision was therefore made not to include demographic factors in the evaluations presented in the following sections.

#### 3.2.2. Comparisons of the Test Variants (Based on Application Type and Input Method)

The following paragraphs address the comparison of the implicit assessments obtained using the four different input methods.

##### 3.2.2.1. D Score Evaluation

D scores between the four test variants, i.e., “native app, keyboard,” “web app, keyboard,” “native app, touch screen,” and “web app, touch screen” do not seem to differ much. Descriptively, independent of the test method applied, there are only insignificant differences between the mean D score values of the four test variants (see Table 3).

**Table 3:** D scores representing implicit preferences vs. combination of app type and input method.

**Implicit ratings**	**Native app +** **keyboard (*N* = 51)**	**Native app + touch** **screen (*N* = 51)**	**Web app + keyboard** **(*N* = 51)**	**Web app + touch** **screen (*N* = 51)**	***P-value***
**D scores**					0.660
Mean (SD)	−0.56 (0.39)	−0.66 (0.35)	−0.60 (0.39)	−0.60 (0.37)	
Range	−1.38 to 0.49	−1.35 to 0.01	−1.50 to 0.31	−1.49 to 0.26	

*For P-value calculation, a linear model ANOVA was used*.

This is to be expected, as D scores are calculated as relative values based on the latencies recorded within each of the four blocks of an IAT test. Consistently longer (or shorter) latencies depending on the input method or application type—which, as the following paragraph will show, are a reality—should therefore not influence the calculated D scores, even though (average) latencies clearly differ. Additionally, in the study, the order in which the four tests were administered to each participant was randomized. There was also a short pause of variable length (usually around one minute) in between the tests. Thus, for the overall group of participants, fatigue due to repeated testing should also not have played a role with respect to the D score calculation (see below for a closer look at the influence of the test order on the results).

##### 3.2.2.2. Evaluation of Latency Values

While there were no significant differences in the calculated D scores between the four test methods, the same does not hold true regarding the (mean) latencies. The results differ significantly, as can be seen in Table 4. Similar to the D score calculations, where latency values below 400 and above 10,000 ms were filtered out, these were removed here as well, thus reducing the number of measurements per combination from the maximum number of 6,120 (51 × 120 per test) to the numbers specified in the respective table columns.

**Table 4:** Latency value based comparison of app types and input methods.

	**Native app +** **keyboard (*N* = 6,014)**	**Native app + touch** **screen (*N* = 6,002)**	**Web app + keyboard** **(*N* = 6,029)**	**Web app + touch** **screen (*N* = 6,117)**	***P*** **-value**
**Latency**					<0.001
Mean (SD)	865.1 (540.5)	874.8 (514.0)	917.6 (618.7)	1011.4 (556.8)	
Range	400.1 to 8059.9	400.2 to 8554.5	401.0 to 9325.0	402.0 to 9572.0	

*Linear model ANOVA was used for P-value calculation*.

The data suggests that, at least descriptively, on average, keyboard inputs tend to be faster (i.e., to have a lower latency) than when a touch screen interface is used. Considering mean latencies, there also appears to be a noticeable difference between using the app and the web-based versions of the test. However, as the order in which the tests were performed was randomized for each participant, this warrants additional investigations (see below).

##### 3.2.2.3. Susceptibility to Errors Depending on App Type and Input Method

It was also of interest to what extent the input mode or program type being used had an influence on the number of errors the users made when performing the four tests. Descriptively, there appear to be slightly more mistakes on average for the web-based app, although the differences between the four combinations of app type and input method are statistically insignificant (*P* = 0.733, Table 5).

**Table 5:** Average number of errors per participant vs. test type.

	**Native app +** **keyboard (*N* = 51)**	**Native app + touch** **screen (*N* = 51)**	**Web app + keyboard** **(*N* = 51)**	**Web app + touch** **screen (*N* = 51)**	***P*** **-value**
**Number of errors**					0.733
Mean (SD)	6.4 (5.7)	5.9 (4.5)	7.0 (5.3)	6.6 (5.1)	
Range	0.0 to 28.0	0.0 to 25.0	0.0 to 22.0	0.0 to 22.0	

*P-value calculation is based on a linear model ANOVA*.

#### 3.2.3. Comparison Based on the Order of the Four Tests

##### 3.2.3.1. Proper Randomization of Test Order vs. Test Type

For evaluating the data with respect to the order in which the tests were performed per participant, it was first of interest whether there was adequate randomization. Table 6 shows the distribution of the four variants vs. the order in which the tests were taken. There was no significant dependency (*P* = 0.752) between the type of test and the order in which the tests were administered. Thus, randomization was satisfactory.

**Table 6:** Test variants vs. order in which they were conducted (random assignments per participant).

	**Test 1 (*N* = 51)**	**Test 2 (*N* = 51)**	**Test 3 (*N* = 51)**	**Test 4 (*N* = 51)**	***P*** **-value**
**Test variant**					0.752
App keyboard	18 (35%)	9 (18%)	12 (24%)	12 (24%)	
App touch screen	11 (22%)	14 (27%)	14 (27%)	12 (24%)	
Web keyboard	13 (25%)	13 (25%)	11 (22%)	14 (27%)	
Web touch screen	9 (18%)	15 (29%)	14 (27%)	13 (25%)	

*P-value calculation is based on Pearson's Chi-squared test*.

##### 3.2.3.2. D Score Evaluation

Descriptively, there does seem to be a small trend in D scores and corresponding ratings depending on the order in which the tests are performed, independent of the type of test that was taken. However, although mean D scores slightly decrease with each additional, this is not statistically significant (*P* = 0.096), as can be seen in Table 7.

**Table 7:** D scores representing implicit preferences vs. test order.

	**Test 1** **(*N* = 51)**	**Test 2 (*N* = 51)**	**Test 3 (*N* = 51)**	**Test 4 (*N* = 51)**	***P*** **-value**
**D scores**					0.096
Mean (SD)	−0.71 (0.36)	−0.60 (0.39)	−0.55 (0.32)	−0.55 (0.42)	
Range	−1.50 to 0.26	−1.49 to 0.31	−1.23 to 0.10	−1.43 to 0.49	

*P-value calculation is based on a linear model ANOVA*.

##### 3.2.3.3. Evaluation of Latency Values

As shown in Table 8, for the latency values, the order in which the tests are being administered is however important (*P* < 0.001). This holds true independent of which kind of test combination (i.e., native app or web-based testing, using either keyboard or touch screen input) is being applied. As practice increases, the participants' measured latencies decrease. Similar to the D score calculation, where latency values below 400 ms and above 10,000 ms were filtered out, these were removed here as well, thus reducing the number of measurements per combination from the maximum number of 6,120 (51 · 120 measurements per test) to the numbers presented in Table 8.

**Table 8:** Test order vs. latency measurements.

	**Test 1 (*N* = 6,067)**	**Test 2 (*N* = 6,053)**	**Test 3 (*N* = 6,050)**	**Test 4 (*N* = 5,992)**	***P*** **-value**
**Latency**					<0.001
Mean (SD)	1014.3 (670.3)	922.2 (522.7)	876.9 (516.9)	856.5 (507.4)	
Range	400.3 to 9572.0	400.3 to 6126.0	400.2 to 8554.5	400.1 to 8218.0	

*P-value calculation is based on a linear model ANOVA*.

The data thus supports the assumption that overall, there is indeed a dependency of the latency values measured in the trials on the order of tests: For the later tests, the measured latencies are on average lower than for the earlier tests. This may reflect the increasing experience of the participants in performing the tests multiple times—even if the input methods and app types differ—as well as familiarization effects with respect to the IAT itself.

##### 3.2.3.4. Susceptibility to Errors Depending on Test Order

Similar to the type of test being applied, there was no apparent influence regarding the average number of errors per test with respect to the order in which the tests were taken (Table 9, *P* = 0.85).

**Table 9:** Distribution of the number of errors per participant vs. test order.

	**Test 1 (*N* = 51)**	**Test 2 (*N* = 51)**	**Test 3 (*N* = 51)**	**Test 4 (*N* = 51)**	***P*** **-value**
**Number of errors**					0.850
Mean (SD)	6.0 (5.1)	6.9 (5.5)	6.4 (4.9)	6.6 (5.2)	
Range	0.0 to 22.0	0.0 to 22.0	0.0 to 25.0	0.0 to 28.0	

*P-value calculation is based on a linear model ANOVA*.

#### 3.2.4. *Post-hoc* Power Calculation

*Post-hoc* power analysis of the linear ANOVA showed 0.8 by *f*^2^=0.25 α=0.05, and four predictors.

## 4. Discussion

### 4.1. Principal Findings

Based on the easily extensible ResearchKit, we were able to create a responsive app that was appropriate for the purposes of the research presented here. Moreover, feedback from the participants indicated that the app was sufficiently intuitive to use. For others who are interested in using the implicit association test in their own (research) apps, the code of the IAT tasks is available on GitHub (45).

Using the study app, it was possible to show that a mobile, ResearchKit-based implementation of the IAT has the potential to be equivalent to other manners of administering this test. We were unable to find any significant differences between either of the two test methods (established, web-based test method vs. native, ResearchKit-based app) combined with two input methods (touch screen vs. keyboard interfaces): Overall, results for the D scores (and corresponding categories of implicit opinions) did not diverge in a statistically significant manner, and neither did the number of errors change significantly for specific combinations of app type and input method (*P*=0.733) or the test order (*P*=0.85).

Nevertheless, there were relevant differences in latency values (corresponding to the users' reactions to the stimuli they were presented with) for both the combinations of app and input types, as well as for test order (both *P* < 0.001). This is, however, at least in part easily explained:

Regarding application and input type, it is not only the technology in use which may influence the recorded user latency values. Varying response times of the touch display and the keyboard, possibly also differences of input and output delays that may originate in the manner of implementation, including the toolkits being used, may have an impact here. In addition, there are human factors to consider (46), and these may for example be related to differences in posture between using a keyboard or a touch interface when interacting with the app (47), or a user's perception of tactile effects when using the different input methods (46, 48). For the test order, with average latency values decreasing with each additional test, it seems sensible to conclude that faster response times may be due to increasing practice. Nevertheless, the differences that were noted regarding latencies do not seem to have influenced the calculated D score values and corresponding scores. This may be due manner in which D scores are calculated: As long as latency ranges overall stay in sync for a single test, the influence on the D scores' calculation, which is essentially based on a ratio between the values of individual test blocks, should be negligible.

As such, our results support the idea, that for the iOS platform, ResearchKit seems well suited for implementing various kinds of research related apps [also see, for example, (17, 19, 49–52)], be it for use in a laboratory setting or for research conducted in field studies. This may also extend to similar libraries on other mobile platforms as well.

There are however several considerations and limitations to be kept in mind that specifically relate to the implementation of the study presented here (see below), as well as the IAT itself, and its implementation.

#### 4.1.1. Development Related Considerations

Standardized frameworks such as ResearchKit (53) or even the PHP and JavaScript-based framework (20, 22, 27) that the web application employed in the study was based on, may well be able to facilitate the development of apps to be used in scientific research. Predefined modules such as surveys, consent, and active tasks can be designed, connected, configured, and filled with appropriate content, e.g., informative descriptions and answer options. However, apps built using any type of framework may be required to follow a certain, predefined “look and feel” that may not be fully adjustable to one's desires. This may for example relate to the use of specific styles and layouts for interactive elements such as buttons that a user may interact with. In the case of the two app types compared in the study, this was a concern: We first suspected that differences in the size of the touch buttons, whenever the touch interface was used (i.e., much smaller, round buttons for the native app vs. control elements encompassing a larger area on either side of the screen for the web-based version) might have influenced the measurements, as we thought that the larger elements for the web solution would have been more forgiving with respect to triggering the respective (correct) touch event. However, as seen in Table 4, this was not supported by the average latencies that were recorded: rather, the web-based version was slower in this regard. A possible explanation for this effect might be that, while the PHP part of the web-based app was of course already interpreted by the web server, the JavaScript code still needed to be interpreted in the devices' web browser. This, along with the inherent latencies of the browser interface itself, might have slowed down the interaction, compared to the natively running code for the ResearchKit-based app, with latency values increasing accordingly. We did, however, not actually measure these effects. Since the slowdown was presumably constant over the entire test run, we do not believe that the calculated D scores were affected.

Also, building an app-based on such libraries still has to be done programmatically, thus preventing those unfamiliar with app programming for the respective platform from building their own apps. In contrast, a graphical user interface (GUI), allowing for “drag and drop” building of such apps and providing research data like questions and IAT data in an XML-based format (27), would enable researchers to create research apps more easily. However, a research app does not only consist of user interface elements. While building an app may seem easy when just using predefined steps for consent, surveys and active steps, adding functionality going beyond the predefined possibilities may require significantly more effort. This may for example be the case when underlying platform features (such as notifications or reminders) are required, or if there is a need to use a participant's location data to determine his or her geographical area. Additionally, adequate methods and procedures for making the results available for evaluation have to be implemented. For this purpose, it is commonly necessary to adapt one's evaluation procedures to varying data formats.

For example, in ResearchKit, all steps—not only surveys and active tasks, but also consent and informational steps—return a nested result object structure. Therefore, one must traverse through a tree structure that reflects the entire process of running the application. As such, the data does not only encompass the data relevant to the test, but also additional meta data.

An additional problem for researchers interested in building their own research apps, be it for IAT testing or different purposes, is that the official ResearchKit framework provided by Apple still only supports development for iOS-based devices (i.e., iPhones, iPads, and iPod touch devices) and the Apple Watch. If one were to use devices based on another (mobile) platform for research, this would necessitate an additional implementation on that platform, possibly requiring a complete redesign for that platform. While there are a number of toolkits available that aim at providing a basic compatibility to ResearchKit, for example, for developing Android-based devices, not all of these are well maintained, and neither do they currently provide the full functionality of ResearchKit. An example of this is the ResearchStack project (15), maintained by Cornell Tech's Small Data Lab and Open mHealth, that closely follows Apple's ResearchKit programming interfaces (APIs) and strives to assist with porting ResearchKit-based apps.

Additionally, aside from the storage aspect itself, for the ResearchKit-based IAT implementation presented here, results are (temporarily) stored in the step results^13^, to be accessed when a task^14^ completes. In theory, after completion of all tasks, it would seem reasonable for the acquired data to be used to calculate the implicit preference of the test subject based on the D score algorithm, and to provide users with appropriate feedback regarding their implicit preferences. However, as mentioned above, this functionality is not included in the ResearchKit IAT implementation.

Apart from these more generic concerns, there are also a few additional points to consider regarding the chosen approach. These deal with the manner of implementation as well as usability and styling related questions.

##### 4.1.1.1. Implementation of the IAT

Aside from instruction and completion steps, in ResearchKit, active tasks commonly only include one active step, often only used once while the task is executed. In contrast, the IAT implementation is more complex and uses its active step^15^ seven times—once per block (*B*_1_ to *B*_7_). In addition, each block has its own instruction step^16^. Furthermore, there are additional steps before the IAT test itself is started, such as an instruction step specifically addressing categories^17^ and a general instruction step. Altogether, there are 17 steps inside the task. Moreover, the task includes the logic for randomizing and shuffling the concept and attribute stimuli, and for creating the appropriate sorting and pairing blocks within the respective trials. As, following ResearchKit's approach, all active tasks are specified in a single file, this is not easy to manage: solely based on the number of steps and the configuration logic, there are more than 3,000 lines of code. This can significantly complicate future maintenance of the code should ResearchKit's APIs introduce breaking changes. For sustainability, it would therefore be desirable to recruit additional programmers for the project who would then participate in the ongoing maintenance of the code.

##### 4.1.1.2. Usability and Technical Peculiarities of the Mobile Interface

In contrast to other active steps that are available in ResearchKit, IAT-based active steps place significantly higher demands on the screen layout with respect to visual components that need to be shown concurrently. For implementations on the web that usually run on larger scale (computer) screens, such as the one provided by Project Implicit (20), this is much less of a problem than when a small mobile device such as a smartphone is used.

For example, the category names of concepts as well as of attributes have to be positioned in the upper left and right part of the screen. A term, which may not only consist of a word, but also an image (both representing the respective concept stimuli), has to be shown, ideally at the screen's center. Last but not least, for recording a user's reaction, two buttons need to be provided on the centered left and right side of the screen, and these buttons are under the constraint that they may not be positioned at the outermost edges of the display. This is to enable users to still hold the devices in their hands, without inadvertently triggering the buttons—a danger especially present on devices with curved displays—while also allowing for a one-handed usage approach, i.e., accessing the buttons with the thumb. Moreover, stimuli must not be too long (for word stimuli) or wide (for image stimuli) in order to still fit between the buttons shown on the screen's left and right side. Additionally, for incorrect responses, a red × needs to be presented, and, while this error indicator is shown, there needs to be a text element instructing the user to select the other button instead. None of these elements may overlap in order to make it possible for users to still correctly trigger touch events on the respective buttons. Especially on smaller iOS-based devices such as iPhone and iPod touch models with a screen diagonal of 4.7 inches, this only leaves very little screen space to work with. When longer instructions are shown to users of such devices, the available space may make it necessary to scroll inside the respective text area to see the stimuli shown on the lower side of the screen. Additionally, as instructions for each trial on the web are presented inside the administration screen of the IAT, they have to be moved to an extra screen prior the actual trial: especially on smart phones, there will otherwise often be insufficient space on the screen. This may negatively impact the user experience.

Also, on current iPhone or iPod touch devices, it is currently impossible to administer the IAT in landscape view, as there would then be insufficient vertical space to prevent UI controls from overlapping. This is however not only a limitation for the IAT presented here, but also for other types of active tasks (e.g., as they are available in ResearchKit) if these are executed on such devices, even if they use fewer UI controls.

#### 4.1.2. Styling

ResearchKit provides a consistent (somewhat fixed) design for surveys, consent and active steps and other elements to allow developers to focus on the actual implementation of a research app by using the templates provided by ResearchKit. This makes it more difficult when custom styling is needed, e.g., for the IAT category and button side names in instruction steps.

### 4.2. Limitations

#### 4.2.1. Recruitment and Study Size

Since we wanted to obtain standardized data for all participants, even going so far as to require that all participants use the same iPad model (and external keyboard), it was necessary to conduct the tests in person.

Due to the ongoing pandemic with its varying contact restrictions, combined with the aforementioned desire to ensure standardization, it was therefore only possible to recruit a limited number of *N* = 56, and complete data sets were unfortunately only obtained for 51 individuals.

*Post-hoc* power analysis nevertheless showed a sufficient power of 0.8 for the linear model ANOVA tests. However, a larger number of participants, ideally from a more diverse background, would have been desirable.

#### 4.2.2. Data Evaluation

Calculating the D scores in a reliable manner relies on appropriate data cleaning procedures, i.e., removing latency measurements that are either too long or too short, perhaps indicating that the respective participant was either distracted or possibly unintentionally triggered a response before the actual classification was made. For the presented evaluation, we decided to retain the established cut-off values for the time being (33), thus removing latencies below 400 or above 10,000 ms. Due to the dependency of the latencies on the app type (native vs. web) and input type (touch screen vs. keyboard) as well as the order of the test execution, one should consider whether it would make sense to optimize this in the future. However, the extent of the data recorded in this study was too small to be able to make a statement on this.

#### 4.2.3. Follow Up

Since the datasets for the participants were only recorded in one session per individual and there was no second appointment, no statement can be made as to whether the participants (either individually or as a group) would also have shown similar test results for the mobile or web-based IAT implementations over a longer period of time.

However, this study was not concerned with an evaluation of the longer-term stability of the test procedure or the IAT *per se*. Rather, it was meant to establish basic comparability of the newly designed native implication based on ResearchKit with another, already established web-based implementation, which we believe to have accomplished.

### 4.3. Future Work

The results of the presented work point to at least basic comparability of the ResearchKit-based IAT implementation to existing approaches [more specifically, the web-based version provided by (20, 22, 27)]. Future research will focus on evaluating our approach in a more realistic setting, with a more diverse study population. It is planned to recruit potential participants at a professional conference (e.g., one about diabetes or adipositas related issues) and to invite the attendees to use the IAT app for a topic related to the conference's focus (such as weight bias).

Ideally, the topic chosen for this more detailed evaluation should be one for which Project Implicit has already acquired and published data for a large number of participants. Many of the published datasets are provided separately on a per-country basis, since social norms and potential biases for specific subject areas may differ in this regard. As this IAT data is commonly available on Open Science Framework's IAT repository (27), the data of our ResearchKit-based app can then be compared to this to determine whether the group of professionals at the chosen conference differs from the (much larger) “Project Implicit” population.

## 5. Conclusions

Despite the limitations of ResearchKit and the current implementation of the IAT, we were able to show at least basic suitability and comparability for administering mobile tests, and, more specifically, for those based on ResearchKit, in the social sciences and psychology. Based on the presented implementation of the IAT, researchers—or their IT staff—may easily build their own IAT-based app.

In settings similar to the one described here, ResearchKit does not only allow the IAT to be used in its predefined form, but also provides researchers with the means to adapt the provided active task to their specific requirement. This may even include building an entirely new version of the tasks, either through appropriate parameterization or by subclassing the provided classes. Altogether, this is a good representation of Apple's statement that ResearchKit “[…] allows researchers and developers to create powerful apps […]” (53), and we expect this approach to be readily transferable to other tests as well. Nevertheless, close collaboration of both researchers and developers remains essential for this to be successful.

## Data Availability Statement

The raw data used in the evaluation will be made available by the authors upon reasonable request. The fork of the base ResearchKit functionalities upon which the app used in the study was based is available on GitHub (45). The fork uses the same BSD style license as ResearchKit itself (54).

## Ethics Statement

For the evaluation part of the work presented here, approval was obtained from the local Ethics Committee of Hannover Medical School (study number 8142_BO_K_2918, dated 05.11.2018).

## Author Contributions

TJ was responsible for programming the ResearchKit-based functionality as well as the app used in the evaluation, recruited the volunteers to be used in the preliminary evaluation and administered the tests. All authors discussed the GUI aspects of the app design. U-VA conceived the part of the study presented here and all authors participated in designing the survey as well as the overall study. UJ adapted the web-based version of the test used in the evaluation and deployed it on the web server. All authors discussed and contributed to the evaluation of the collected data, contributed to writing the manuscript, and approved the submitted version.

## Funding

We acknowledge support by the German Research Foundation (DFG) and the Open Access Publication Fund of Hannover Medical School (MHH) for the publication fees.

## Conflict of Interest

The authors declare that the research was conducted in the absence of any commercial or financial relationships that could be construed as a potential conflict of interest.

## Publisher's Note

All claims expressed in this article are solely those of the authors and do not necessarily represent those of their affiliated organizations, or those of the publisher, the editors and the reviewers. Any product that may be evaluated in this article, or claim that may be made by its manufacturer, is not guaranteed or endorsed by the publisher.

## Footnotes

^1^Throughout the text, wherever “iOS” is used, this encompasses both the iOS platform for iPhone and iPod touch devices as well as the iPadOS platform for iPads.

^2^For example, ORKOrderedTask or ORKNavigableOrderedTask implementing ResearchKit's ORKTask protocol.

^3^Subclassed from ORKStep.

^4^For example, ORKQuestionStep for a single question and answer pair, or ORKFormStep for forms with multiple elements, e.g., for asking participants about their, name, date of birth, or other information on a single screen.

^5^Subclassed from ORKActiveStep.

^6^Derived from ORKTaskViewController.

^7^Derived from ORKStepViewController.

^8^That is, ORKImplicitAssociationStep, ORKImplicitAssocia-tionContentView, ORKImplicitAssociationStepViewControl-ler, and ORKImplicitAssociationResult.

^9^ORKImplicitAssociationCategoriesInstructionStep, ORKIm-plicitAssociationTrial, and ORKImplicitAssociation-Helper.

^10^ORKHeadlineLabel and UIImageView, respectively.

^11^based on ORKImplicitAssociationCategoriesInstructionStep and ORKInstructionStep, respectively.

^12^ORKCompletionStep, with the option to skip this via ORKPredefinedTaskOptionExcludeConclusion.

^13^ORKStepResult.

^14^that implements the ORKTask protocol.

^15^ORKImplicitAssociationStep.

^16^ORKInstructionStep.

^17^ORKImplicitAssociationCategoriesInstructionStep.
